# Interventions for breastfeeding-related nipple pain or injury: a meta-analysis

**DOI:** 10.3389/fgwh.2025.1507723

**Published:** 2025-07-09

**Authors:** Xuyan Jia, Yang Dong, Cuili Shen, Yuanyuan Cai, YiTing Xu, Lina Yang, Jiayue Jiang, Tong Sun, Wenhui Lu, Rong Huang

**Affiliations:** ^1^ School of Medicine, Tongji University, Shanghai, China; ^2^ Songjiang Maternal and Child Health-care Hospital, Shanghai, China; ^3^ Women and Children Society of Health & Education of Putuo District, Shanghai, China; ^4^ Outpatient and Emergency Office, Shanghai Municipal Hospital of Traditional Chinese Medicine, Shanghai University of Traditional Chinese Medicine, Shanghai, China; ^5^ Shanghai First Maternity and Infant Hospital, School of Medicine, Tongji University, Shanghai, China

**Keywords:** nipple, pain, injury, early medical intervention, breastfeeding

## Abstract

**Background:**

Nipple pain or injury is one of the main reasons many mothers stop breastfeeding. We integrated existing literature and conducted a quantitative evaluation of efficacy, with the aim of identifying effective clinical interventions for alleviating breastfeeding-related nipple pain and injury, and providing evidence-based recommendations for future research and clinical practice. In our study, non-specific interventions are defined as measures that do not specifically provide breastfeeding support and are designed to exclude natural factors or induce placebo effects. Conversely, interventions that target the study outcomes and have standardized operational procedures are referred to as specialized interventions.

**Methods:**

We conducted a systematic literature search across 9 databases, including MEDLINE (via Ovid), PubMed, Web of Science, CINAHL (via EBSCO), EMBASE, the Cochrane Library, CNKI, SinoMed, and Wanfang. Two independent reviewers screened the publications and extracted the data. We evaluated the quality of literature on randomized controlled trials (RCTs) and quasi-experimental clinical trials using the Cochrane Systematic Evaluation Risk of Bias Assessment Tool and the JBI Critical Appraisal Tool. After completing the quality assessment of the literature, we performed a meta-analysis using Stata 17.0.

**Results:**

A total of 18 studies were identified in the meta-analysis. The results of the meta-analysis showed that specialized interventions were significantly better than non-specific interventions in preventing and treating nipple pain and injury associated with breastfeeding. The specialized interventions were effective in reducing the incidence of nipple pain [OR = 0.366, 95% CI (0.155, 0.862), *Z* = −2.301, *p* = 0.021 < 0.05], nipple pain scores [SMD = −0.451, 95% CI (−0.748, −0.154), *Z* = −2.978, *p* = 0.003 < 0.05], incidence of nipple injury [OR = 0.316, 95% CI (0.231, 0.433), *Z* = −7.177, *p* < 0.001], and intensity of nipple injury [SMD = −0.964, 95% CI (−1.404, −0.525), *Z* = −4.303, *p* < 0.001].

**Conclusion:**

This study shows that specialized interventions for breastfeeding-related nipple injury and pain are significantly more effective than non-specific interventions. It also demonstrates that preventive measures initiated before nipple pain onset are more effective than post-pain interventions.

**Systematic Review Registration:**

https://www.crd.york.ac.uk/PROSPERO/view/CRD420251045411, PROSPERO CRD420251045411.

## 1 Introduction

The World Health Organization (WHO) and the United Nations Children's Fund (UNICEF) recommend that babies should be exclusively breastfed for the first 6 months of life, and that breastfeeding should continue until the infant is 2 years old, with the addition of complementary foods. Breastfeeding is not only good for the health of children but also for that of the mother. For newborns, breastfeeding reduces the risk of necrotising small bowel colitis and advanced sepsis, and contributes to the development of the child's immune system (1, 2). For mothers, there is evidence that breastfeeding reduces the risk of type 2 diabetes, breast carcinoma, ovarian carcinoma, and endometrial cancer (3).

Many new mothers have the intention to breastfeed but give up for various reasons, with nipple soreness being one of the most common, second only to inadequate milk supply (4). The skin of the nipple area is exceptionally sensitive to pain due to its rich nerve endings. The unpleasant sensation of pain directly causes parturients to subjectively shorten breastfeeding duration and reduce feeding frequency (5). Nipple cracking accounts for 26% of cases of early discontinuation of breastfeeding in a US study (<24 weeks' duration) (6). Newby showed that 30.4% of mothers stop breastfeeding within 12 weeks owing to nipples that were sore, cracked or bleeding (7). In China, nipple pain was also one of the reasons for giving up breastfeeding within one month after childbirth, accounting for 19.2% (8).

Postpartum nipple pain is very common. A study in the United Kingdom (UK) showed that 76% breastfeeding women experienced latch-related nipple pain (5). According to Buck, 79% of women reported nipple pain prior to discharge from the hospital. Moreover, at 8 weeks postpartum, 8% of women still had nipple trauma and 20% still had nipple pain (9).

The International Association for the Study of Pain defines pain as “an unpleasant sensory and emotional experience associated with, or resembling that associated with, actual or potential tissue injury.” As also stated in NOTES, “pain may have adverse effects on function and social and psychological well-being” (10). In previous studies, nipple pain has been specifically described as shooting, hot, burning, stinging, tight, and tearing pain (11). Nipple injury, which includes ecchymosis, blisters, and marks, is often accompanied by increased sensitivity of the nipple–areola region or acute pain, especially in early breastfeeding (12).

Current research on interventions for breastfeeding-related nipple pain or injury encompasses a diverse array of approaches, including topical agents, laser therapy, breastfeeding postures, health education, and more. While numerous studies exist, there is no uniform consensus on the effectiveness of these measures. To develop programs to prevent or alleviate breastfeeding-related nipple pain, we conducted a scoping review of previous research findings in the present study and used meta-analysis to assess the effectiveness of various measures. Such findings could be used to help promote breastfeeding.

## 2 Methods

### 2.1 Aim

This study aimed to consolidate existing evidence and conduct a quantitative assessment of intervention efficacy, with the primary objectives to identify effective clinical strategies for alleviating breastfeeding-related nipple pain and injury, and provide evidence-based recommendations for future research and clinical practice.

### 2.2 Study design

The study design adopted a scoping review methodology guided by Arksey and O'Malley (13) and enhanced by Levac and colleagues (14). We used the Joanna Briggs Institute Reviewer's Manual to conduct the review, which involves the following stages: (a) identifying the research question(s), (b) inclusion and exclusion criteria, (c) search strategy,(d) evidence screening and selection,(e)data extraction,(f) data analysis(g) presentation of the results, (h) summarizing and reporting the results (15). This study was conducted following the PRISMA guidelines. The meta-analysis was preregistered at the International Prospective Register of Systematic Reviews (PROSPERO), and the registration number is CRD420251045411 (16).

To answer the research objective, the review questions were as follows: (1) What are the interventions for breastfeeding-induced nipple pain and injury? (2) What is the effectiveness of current interventions for nipple pain and nipple injury?

The PICOS framework (population, intervention, comparison, outcomes, and study designs) was followed. This study will include studies collecting data from participants meeting the following criteria. (1) Population: breastfeeding mothers without abnormal nipple problems, and no oral, palate, or maxillofacial abnormalities in the newborn. (2) Intervention: the article describes interventions to prevent or manage nipple pain and injury. (3) Comparison: the control group was non-specific interventions including application of breast milk, use of placebo and no intervention. (4) Outcomes: study results related to the incidence of nipple pain, degree of nipple pain, incidence of nipple injury, and degree of nipple injury. (5) Study design: we included quantitative and/or qualitative research conducted in both English and Chinese.

The following studies were excluded: (1) literature review articles, case reports, dissertations, and conference papers; (2) studies that do not use an intervention or implementation strategy; (3) studies in languages other than Chinese and English; (4) unable to obtain the full text or required data; (5) newborns with tongue-tie problems; (6) breast abnormalities such as short, flat, and sunken nipples in breastfeeding mothers.

### 2.3 Search strategies

We searched nine databases: MEDLINE (through Ovid), PubMed, Web of Science, CINAHL (through EBSCO), EMBASE, Cochrane Library, CNKI, SinoMed, and Wanfang in January 2024. Searched using a combination of subject terms and free words. The search time frame ranged from January 1, 2019 to December 31, 2023.

The search terms adopted for the review were: nipple pain or nipple trauma or nipple crack or nipple fissure or nipple injury or nipple damage or nipple wound or nipple chap or nipple cleft, assistance or care or control or education or intervention or management or nursing or treatment or cure or method or prevention or step or measure, breast feeding or breastfeeding.

### 2.4 Literature screening and data extraction

Based on predetermined literature inclusion and exclusion criteria, each of these researchers independently screened the literature and extracted information. A third researcher adjudicated when disagreements were encountered. The identified publications were imported into EndNote and duplicates were removed. Initial screening was carried out by reading the title and abstract of each publication. After excluding clearly irrelevant literature, the full text was read to determine study eligibility for final inclusion. Data extracted comprised the author(s), year of publication, type of study, sample size, intervention group, control group, intervention duration, outcomes, whether side effects were reported.

### 2.5 Quality assessment

Quality evaluations were independently conducted by two reviewers. In cases where discrepancies emerged, they were resolved through discussions involving a third reviewer. We appraised the methodological quality of the included randomized controlled trials (RCTs) using the Cochrane Collaboration's Risk of Bias Assessment Tool (17). The Cochrane collaboration's tool for RCTs assesses risk of bias on the following domains: random sequence generation; allocation concealment; blinding of participants and personnel; blinding of outcome assessors; incomplete outcome data; selective reporting and other bias. The judgment for each item in every study was categorized as “low,” “high,” or “unclear” based on the extent of bias present. Regarding the overall quality assessment, studies with a high—risk rating in one or more of these domains were classified as having a high overall risk of bias. When no high—risk items were identified, but one or more items were rated as unclear, the study was considered to have an unclear overall risk of bias. Conversely, studies that were deemed to have a low risk across all domains were categorized as having a low overall risk of bias. The quality of quasi-experimental clinical trials was appraised using the JBI critical appraisal tool (18).

### 2.6 Statistical analysis

The statistical analysis was performed using STATA 17.0. For continuous variables, the standardized mean difference (SMD) with 95% confidence intervals (CI) was used as the effect size. For discontinuous variables, the risk ratio (RR) or odds ratio (OR) with 95% CI was used as the effect size. Cohen's categories were used to evaluate the magnitude of the overall effect size with (1) SMD = 0.2–0.5: small; (2) SMD = 0.5–0.8: medium, and (3) SMD >0.8: large effect sizes (19).

Heterogeneity across study results was evaluated using Cochran's *Q* test and *I*² statistic. When statistical heterogeneity was absent (*p* > 0.10, *I²* < 50%), a fixed—effects model was applied. Conversely, significant heterogeneity (*p* ≤ 0.10, *I²* ≥ 50%) warranted the use of a random—effects model (20).

Sensitivity analyses were conducted using the leave-one-out approach to systematically evaluate the impact of each study on the pooled estimates, thereby ensuring the robustness of our findings. Publication bias was assessed using the PET-PEESE method, visualized through PET regression plots in JASP software, and complemented by funnel plots and Egger's regression test.

## 3 Results

### 3.1 Literature search and screening results

**Figure 1:**
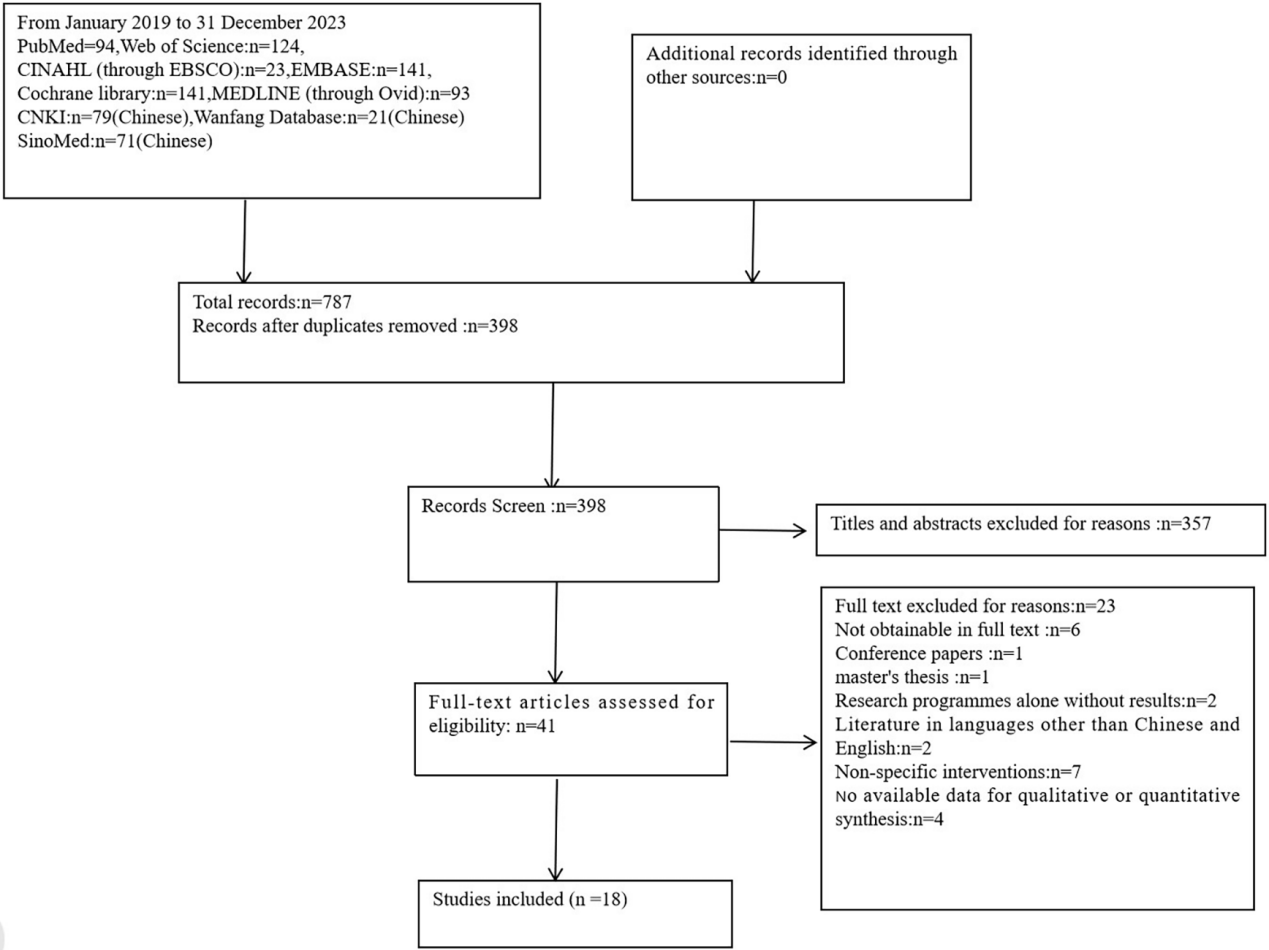
Flow diagram based on PRISMA guidelines.

Using the above search strategy, we identified 787 potential articles in the nine databases searched, of which 389 duplicates were excluded in EndNote. Based on inclusion and exclusion criteria in the PRISMA flowchart, a final 18 articles were included after screening the title, abstract, and full-text (Figure 1).

### 3.2 Basic characteristics of the included studies

A total of 2,250 breastfeeding women were included in the study. 18 included studies were conducted across various countries: Iran (*n* = 6), Turkey (*n* = 4), China (*n* = 3), Brazil (*n* = 2), United States (*n* = 1), Italy (*n* = 1), Bosnia and Herzegovina (*n* = 1). In terms of study design, 17 studies were randomized controlled trials, one was quasi-experimental clinical trial. The 18 studies included 11 studies on topical preparations for the prevention or treatment of nipple pain or injury, specifically beeswax (21), coconut oil or tea tree oil (22), olive oil (23, 24), pure lanolin (25), coconut oil (26), cocoa butter (27), vernix caseosa (28), Achillea millefolium (yarrow) (29), aloe vera gel (30),mountain honey or boiled yarrow (31). Two studies focused on breastfeeding positions, specifically the baby-led self-attachment breastfeeding position (32, 33). Two studies investigated localized physical interventions: breast shells (34) and laser therapy (35). Additionally, three studies examined education-related measures (36–38). The characteristics of the included studies were shown in Table 1.

**Table 1:** Descriptive information for studies included in the meta-analysis.

No.	Author	Year	Country	Research design	Groups		Sample size		Intervention duration (days)	Outcomes	Side effects
					Intervention	Control	Intervention	Control			
1	Serhatlioglu et al. (21)	2023	Turkey	RCT	Beeswax	Usual care	30	30	10	(2, (5)	Not reported
2	Şahin et al. (22)	2023	Iran	RCT	Coconut oil/tea tree oil	Usual care	30/30	30	10	(2)	Not reported
3	Lin et al. (23)	2023	China	RCT	Olive oil	Usual care	40	40	3	(2, (5)	Not reported
4	Perić et al. (25)	2023	Bosnia and Herzegovina	RCT	Pure lanolin	Usual care	83	78	7	(4)	Reporting side effects
5	Alikamali et al. (26)	2023	Iran	RCT	Coconut oil	Usual care	50	48	14	(2, 6)	Not reported
6	Can Gürkan Öet al. (27)	2022	Turkey	RCT	Cocoa butter	Usual care	35	37	10	(1, 5)	Not reported
7	Cecilio et al. (34)	2022	Brazil	Quasi- experimental clinical trial	Breast shells	Usual care	29	33	14	(1, 5)	Reporting side effects
8	Vafadar et al. (36)	2022	Iran	RCT	Breastfeeding technique training	Usual care	37	37	15	(5)	Not reported
9	Gao et al. (37)	2022	China	RCT	Ten online antenatal breastfeeding education sessions	Usual care	182	160	3	(5)	Not reported
10	Sağlık et al. (24)	2021	Turkey	RCT	Olive oil	Usual care	40	40	14	(3, 5)	Not reported
11	Doğan Merih et al. (28)	2021	Turkey	RCT	Vernix caseosa	Usual care	32	32	7	(1, 5)	Not reported
12	Yin et al. (32)	2021	China	RCT	Baby-led self-attachment breastfeeding	Usual care	206	203	180	(1)	Not reported
13	Abdoli et al. (29)	2020	Iran	RCT	Achillea millefolium	usual care	40	40	14	(2, 7)	Not reported
14	Hanieh Alamolhoda et al. (30)	2020	Iran	RCT	Aloe vera gel	Usual care	55	55	14	(3, 5)	Not reported
15	Firouzabadi et al. (31)	2020	Iran	RCT	Mountain honey/boiled yarrow	Usual care	50/50	50	7	(6)	Not reported.
16	Camargo et al. (35)	2020	Brazil	RCT	Laser therapy	Placebo	36	38	1	(2)	Reporting side effects
17	Milinco et al. (33)	2020	Italy	RCT	Biological nurturing	Usual care	90	98	120	(1, 5)	Not reported
18	Lucas et al. (44)	2019	United States	RCT	Breastfeeding self-management(BMS)	Usual care	26	30	42	(2)	Not reported.

Note: (1) Incidence of nipple pain; (2) nipple pain scores (visual analogue scale, VAS); (3) nipple pain scores(numerical rating scale, NRS); (4) nipple pain scores (McGill pain questionnaire); (5) incidence of nipple injury; (6) intensity of nipple injury (store scale); (7) intensity of nipple injury(Amir scale).

### 3.3 Outcomes of literature quality evaluation

**Figure 2:**
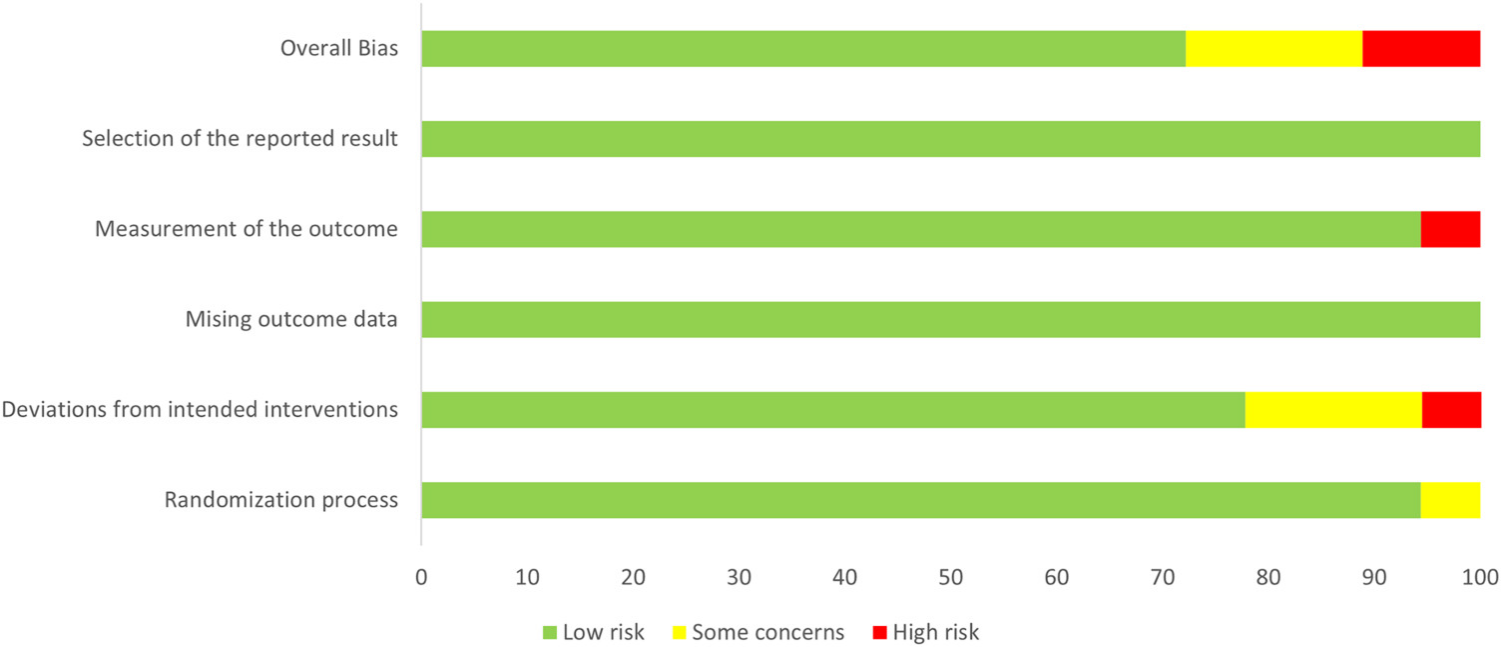
Methodological quality of included studies.

**Figure 3:**
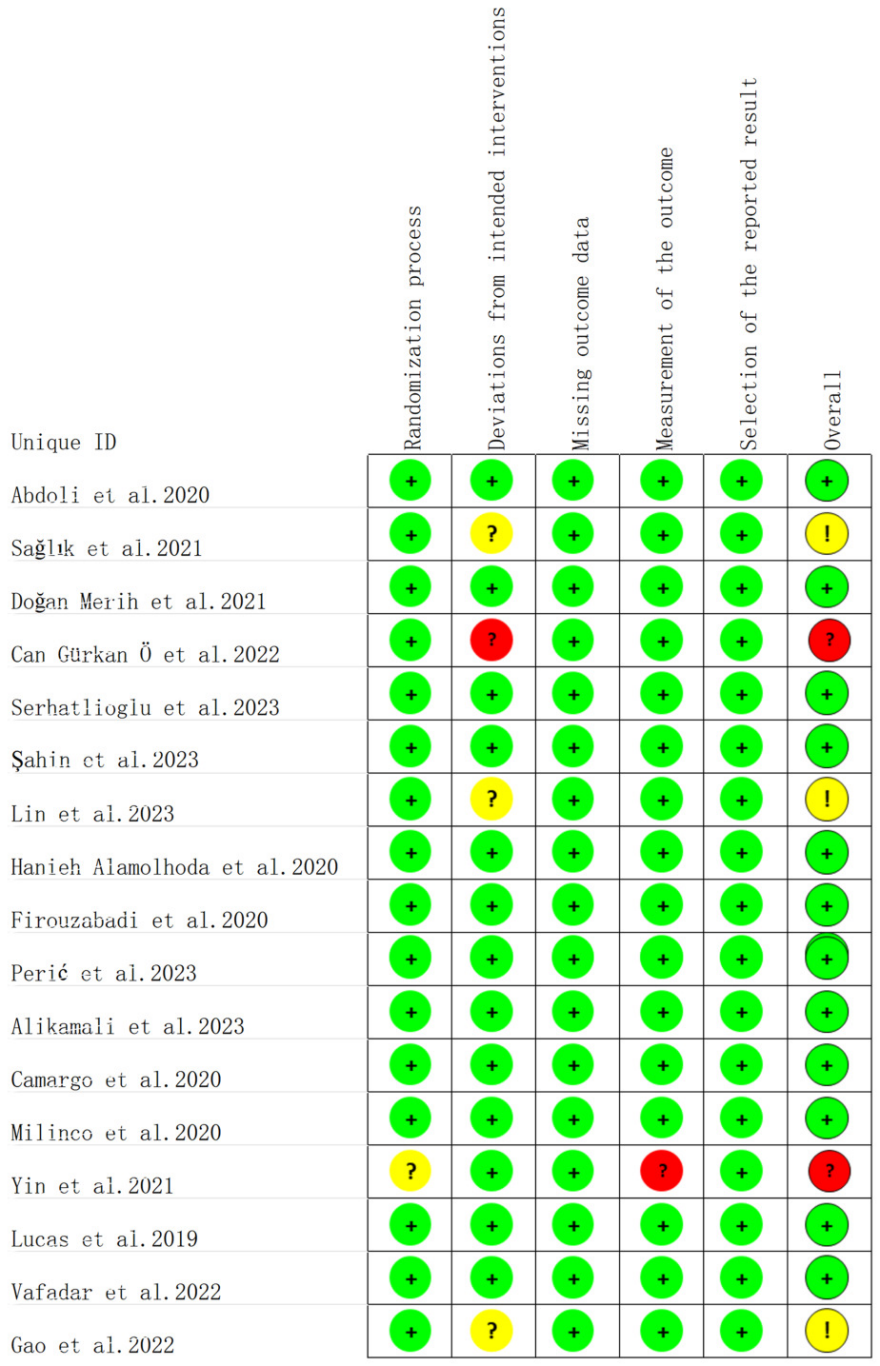
The distribution of the methodological quality of included studies.

The results of the literature quality evaluation showed that among the 17 randomized controlled trials included, 13 studies had a low risk of bias, 3 had a uncertain risk of bias, and 1 had a high risk of bias (Figures 2, 3). All data were kept complete. 5 studies described the detailed allocation concealment (23–25, 33, 35). 4 studies adopted blinding designs, among which 2 studies used single-blind designs (29, 35) and 2 employed double-blind designs (26, 28). A non-randomized controlled trial study was evaluated as low risk (Table 2).

**Table 2:** Quality evaluation of quasi—experimental studies.

Author	Q1	Q2	Q3	Q4	Q5	Q6	Q7	Q8	Q9	%Yes	Risk
Cecilio et al. (34)	Y	Y	Y	Y	Y	Y	Y	Y	Y	100	LOW

Q1. It is clear in the study what is the “cause” and what is the “effect” (i.e., there is no confusion about which variable comes first)?, Q2. Was there a control group?, Q3. Were participants included in any comparisons similar?, Q4. Were the participants included in any comparisons receiving similar treatment/care, other than the exposure or intervention of interest?, Q5. Were there multiple measurements of the outcome, both pre and post the intervention/exposure?, Q6. Were the outcomes of participants included in any comparisons measured in the same way?, Q7. Were outcomes measured in a reliable way, Q8. Was follow-up complete and if not, were differences between groups in terms of their follow-up adequately described and analyzed?, Q9. Was appropriate statistical analysis used?

Y, yes; N, no; U, unclear.

### 3.4 Results of meta-analysis

#### 3.4.1 Effects on the incidence of nipple pain

**Figure 4:**
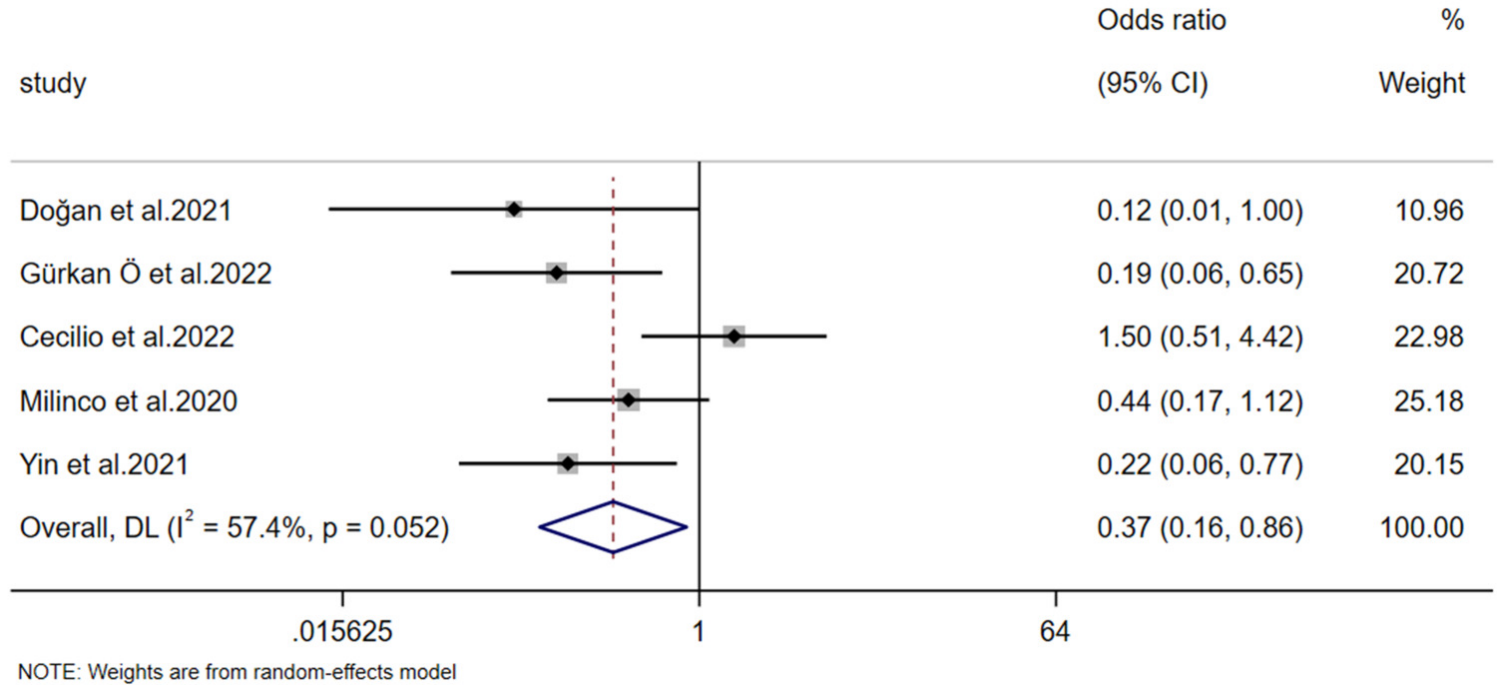
Forest plot of the incidence of nipple pain.

The incidence of nipple pain was reported in 5 studies. A heterogeneity test (*p* = 0.052, *I*² = 57.4%) revealed significant heterogeneity; consequently, a random-effects model was employed for the analysis. Meta-analysis results demonstrated that the experimental group exhibited a significantly better effect than the control group in preventing postpartum nipple pain [OR = 0.366, 95% CI (0.155, 0.862), *Z* = −2.301, *p* = 0.021 < 0.05] (Figure 4).

#### 3.4.2 Effects on the severity of nipple pain

**Figure 5:**
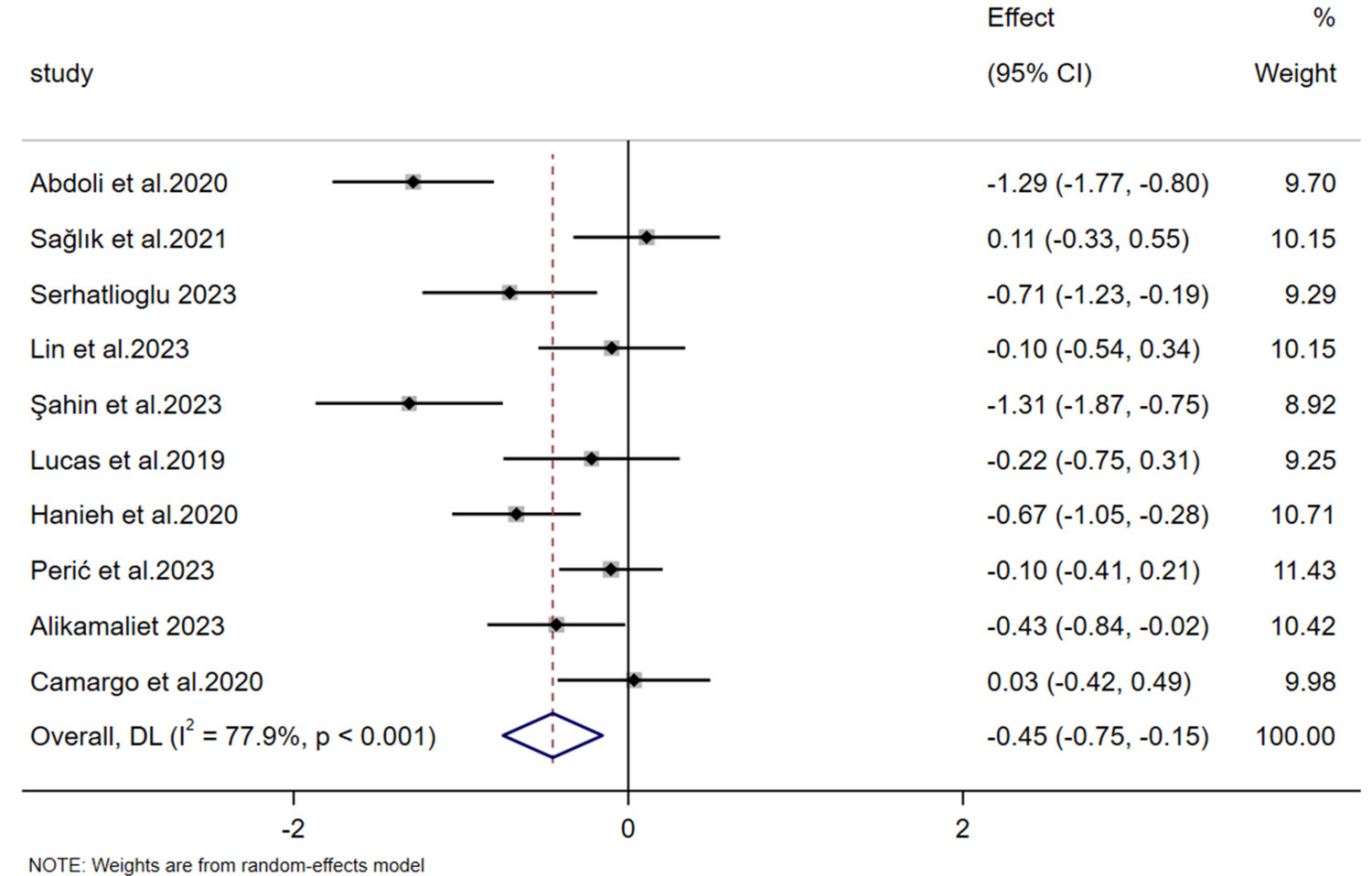
Forest plot of the severity of nipple pain.

The nipple pain scores were reported in 10 studies. The heterogeneity test (*p* < 0.001, *I*² = 77.9%) revealed substantial heterogeneity, prompting the application of a random-effects model for effect-size pooling. Meta-analysis results demonstrated that the experimental group had significantly lower scores for breastfeeding-related nipple pain compared to the control group [SMD = −0.451, 95% CI (−0.748, −0.154), *Z* = −2.978, *p* = 0.003 < 0.05] (Figure 5).

Subgroup analyses were subsequently conducted based on the distinct initiation phases of intervention timing, categorizing each group into an intervention subgroup and a prevention subgroup. Given the significant heterogeneity identified, random—effects models were adopted for both subgroups. For the prevention group, meta-analysis indicated that preventive measures were more effective than the control group in relieving breastfeeding-related nipple pain [SMD = −0.574, 95% CI (−1.070, −0.079), *Z* = −2.273, *p* = 0.023 < 0.05]. In the intervention group, Meta—analysis failed to provide evidence that intervention measures were more effective than the control in alleviating nipple pain [SMD = −0.294, 95% CI (−0.599, 0.011), *Z* = −1.887, *p* = 0.059 > 0.05]. The result in the intervention group (*p* = 0.059 > 0.05) was not statistically significant.

**Figure 6:**
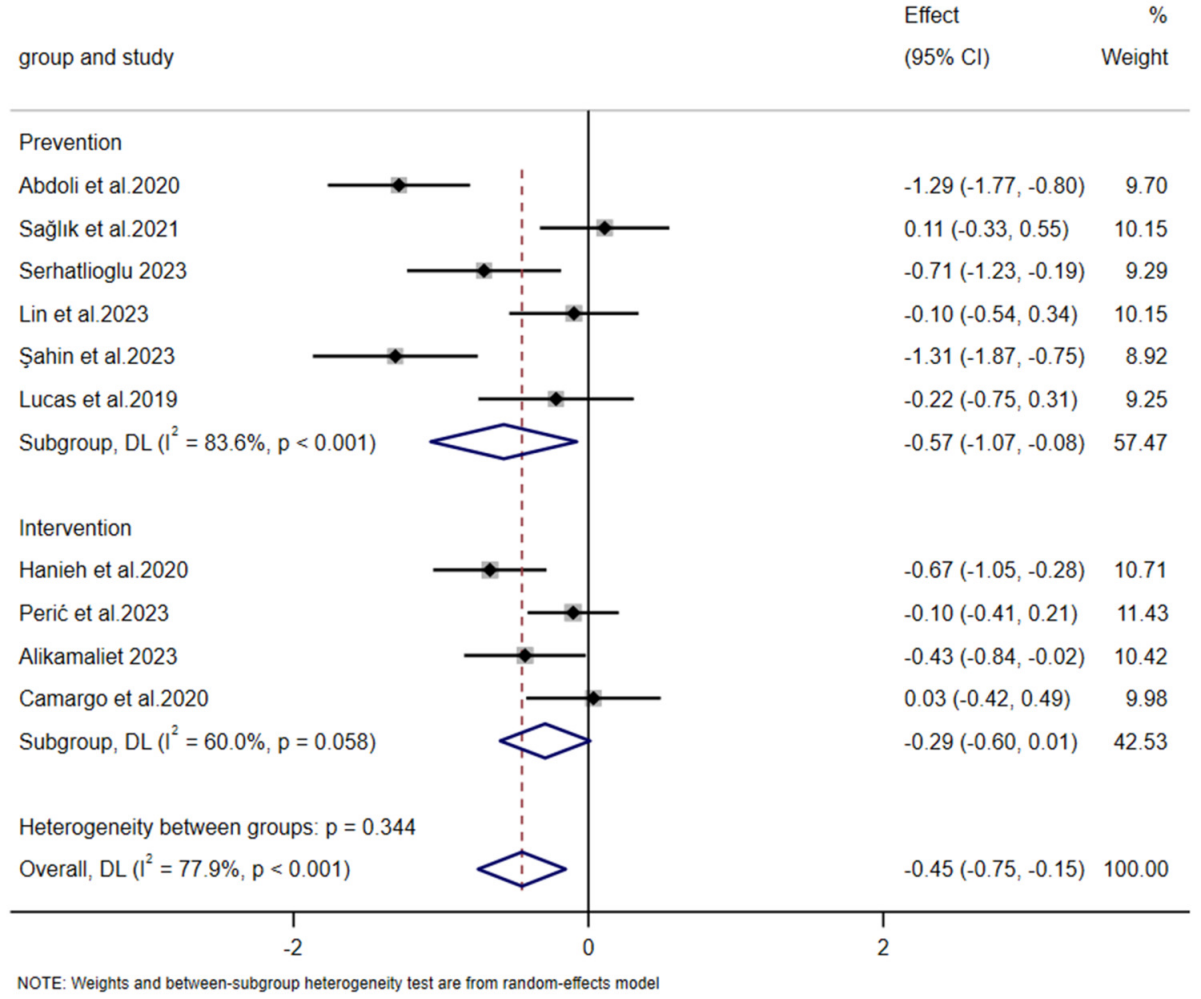
Forest plot of subgroup analysis on the impact of intervention initiation phase on the severity of nipple pain.

**Figure 7:**
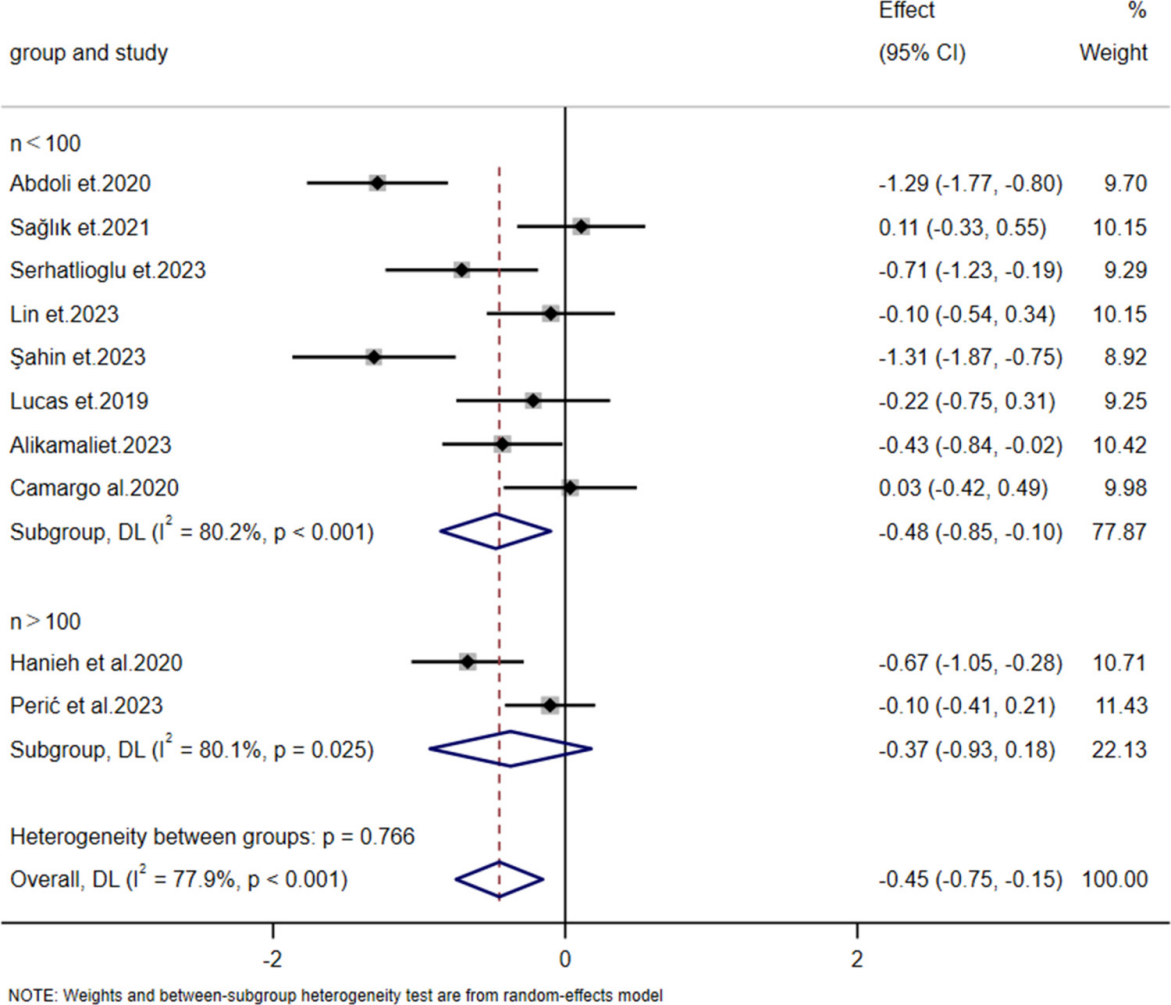
Forest plot of subgroup analysis on the impact of sample size on the severity of nipple pain.

**Figure 8:**
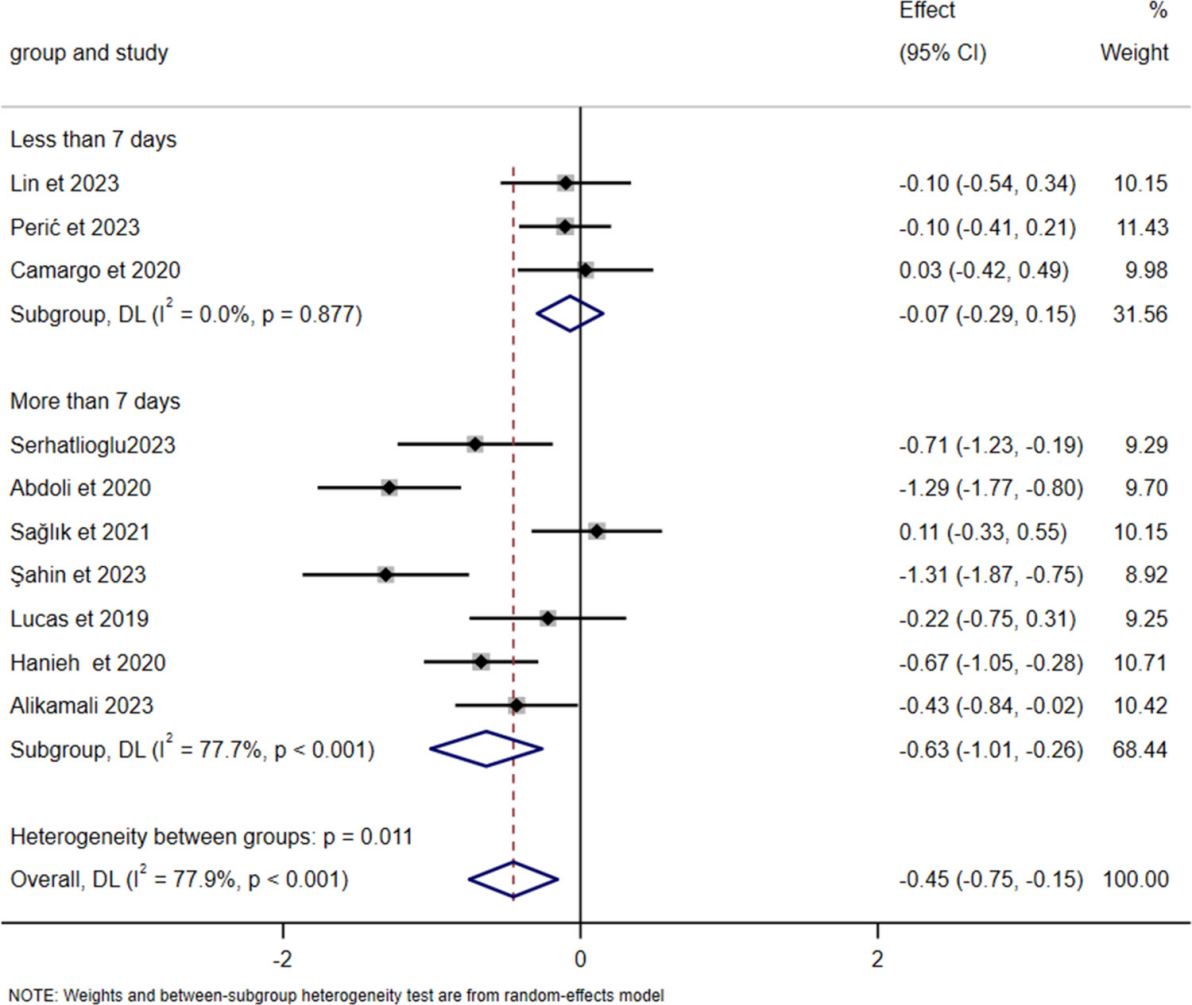
Forest plot of subgroup analysis on the impact of intervention duration on the severity of nipple pain.

There was no significant heterogeneity between the prevention and intervention groups (intergroup heterogeneity: *p* *=* 0.344 > 0.05). Meta-regression analysis showed *p* = 0.427 > 0.05, indicating no statistical significance and suggesting that neither group contributed to the heterogeneity in the meta-analysis (Figure 6). Due to the high overall heterogeneity, we further conducted subgroup analyses based on differences in sample size and intervention duration. The results showed no significant difference across different sample sizes (intergroup heterogeneity: *p* = 0.766 > 0.05, Figure 7). Meta-regression analysis further revealed *p* = 0.821 > 0.05, whereas intervention duration might be a key contributor to the high heterogeneity. In the “Less than 7 days” subgroup, heterogeneity was almost nonexistent (*I*² = 0.0%, *p* = 0.877), indicating high consistency of results across studies with shorter intervention periods (less than 7 days). By contrast, the “More than 7 days” subgroup exhibited substantial heterogeneity (*I*² = 77.7%, *p* < 0.001). The significant difference between subgroups (intergroup heterogeneity: *p* = 0.011 < 0.05) suggests that intervention duration exceeding 7 days profoundly influences the overall study heterogeneity (Figure 8).

#### 3.4.3 Effects on the incidence of nipple injury

**Figure 9:**
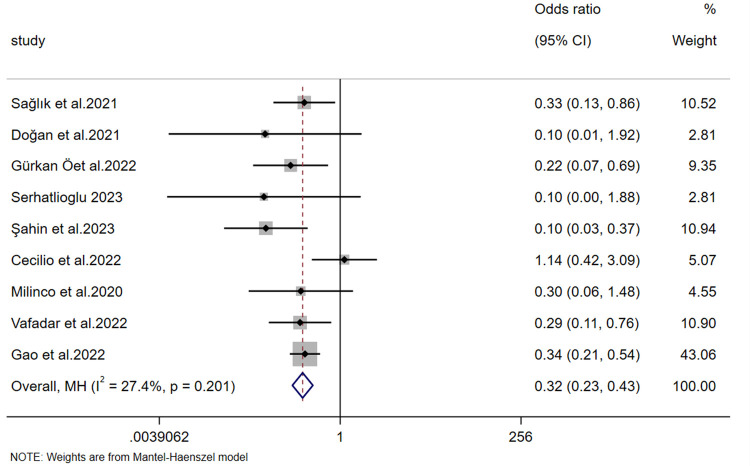
Forest plot of the incidence of nipple injury.

The incidence of nipple injury was reported in 9 studies. The heterogeneity test (*p* = 0.201, *I*² = 27.4%) indicated non-significant heterogeneity; therefore, a fixed-effects model was used to pool the effect sizes. Meta-analysis results showed that the number of women with nipple fissures in the experimental group was significantly lower than that in the control group, demonstrating that the experimental group was significantly more effective in preventing postpartum nipple injury than the control group [OR = 0.316, 95% CI (0.231, 0.433), *Z* = −7.177, *p* < 0.001] (Figure 9).

#### 3.4.4 Effects on the intensity of nipple injury

**Figure 10:**
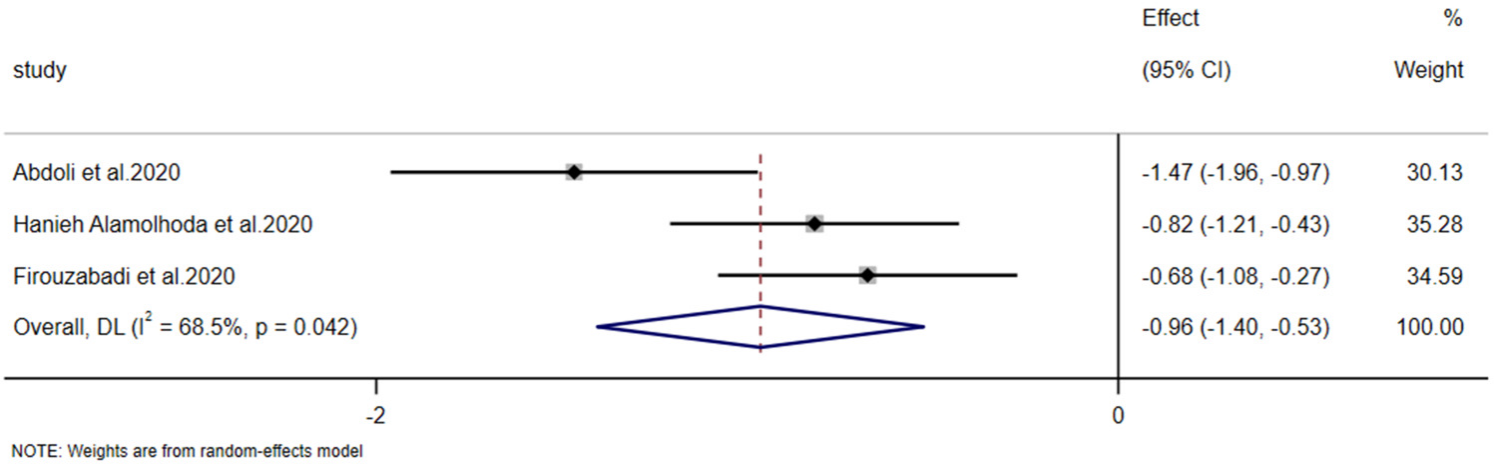
Forest plot of the intensity of nipple injury.

The intensity of nipple injury was reported in 3 studies. The heterogeneity test (*p* = 0.042, *I*² = 68.5%) indicated significant heterogeneity; thus, a random-effects model was used to combine the effect sizes. The results of the meta-analysis showed that the improvement in postpartum nipple injury in the experimental group was significantly greater than that in the control group [SMD = −0.964, 95% CI (−1.404, −0.525), *Z* = −4.303, *p* < 0.001] (Figure 10).

#### 3.4.5 Report on the risk of side effects

**Figure 11:**
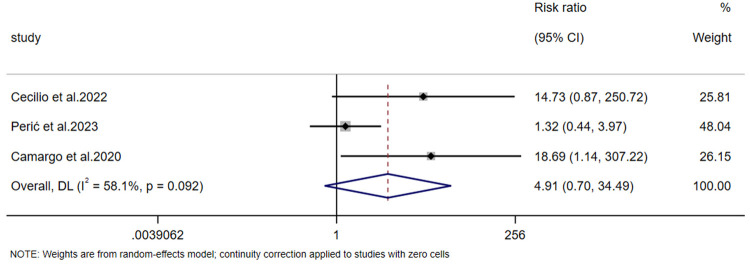
Forest plot of the risk of side effects.

Regarding the risk of side effects, data were reported in 3 studies. The heterogeneity test (*p* = 0.092, *I*² = 58.1%) indicated significant heterogeneity, so a random-effects model was used to pool the effect sizes. The results of the meta-analysis showed that although the risk ratio (RR) suggested a trend of increased side effect risk in the experimental group, there was no significant difference in side effects between the two groups [RR = 4.913, 95% CI (0.700, 34.494), *Z* = 1.601, *p* = 0.109 > 0.05] (Figure 11).

#### 3.4.6 Publication bias assessment

**Figure 12:**
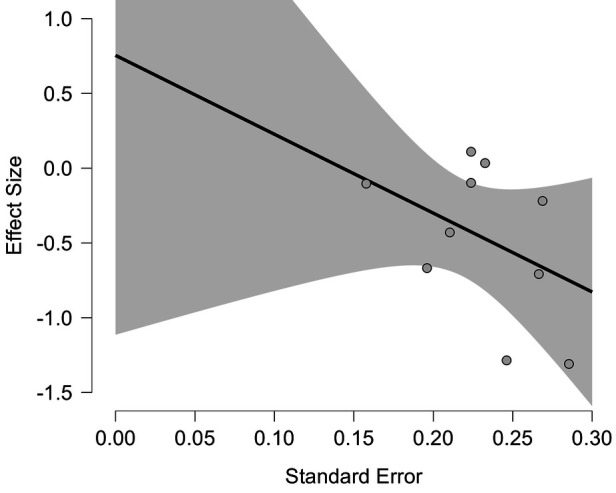
PET regression plot.

We performed PET-PEESE and Egger's test to assess publication bias for the severity of nipple pain, an outcome indicator based on 10 included studies. In the PET—PEESE analysis, the estimated value of the PET method was 0.755 (SE = 0.810, *t* = 0.931, *p* = 0.379 > 0.05). The estimated value of the PEESE method was 0.227 (SE = 0.433, *t* = 0.526, *p* = 0.613 > 0.05). Based on this, we conclude that there is no obvious publication bias in the effect sizes analyzed, and the visual presentation of the PET regression plot further validates this conclusion (Figure 12). The Egger's test results indicated that there was no obvious evidence of publication bias (*p* = 0.189 > 0.05). Neither method detected evidence of publication bias.

**Figure 13:**
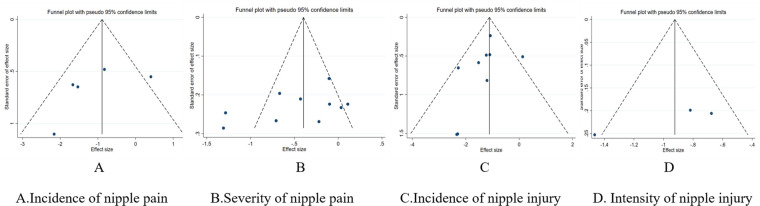
Inverted funnel plot of the assessment of publication bias risk.

Funnel plots were used to assess the bias risk of primary outcome indicators. The results showed that the funnel plots of nipple pain incidence, nipple injury incidence, and nipple injury severity presented uneven distributions, suggesting potential publication bias, which may be related to the small number of included studies (Figure 13). Sensitivity analysis was conducted using the one-by-one exclusion method. The sensitivity analysis results indicated that after excluding the included studies one by one, there were no statistically significant changes in the results, which demonstrated good stability of the findings.

## 4 Discussion

The etiology and management strategies for nipple pain and injury during breastfeeding continue to be highly debated topics within the medical and lactation research communities. Despite decades of clinical experience and research endeavors, there remains a lack of consensus on the effective preventive and therapeutic approaches, underscoring the urgent need for comprehensive evidence synthesis. Therefore, we conducted an in-depth scoping review and meta-analysis, identifying 18 studies aimed at evaluating the efficacy of diverse interventions for preventing or alleviating breastfeeding-associated nipple pain and injury.

The first finding of our study was that a specialised intervention strategy was substantially superior to non-specific interventions in the management of breastfeeding-related nipple pain and injury. This finding was supported by a variety of outcome metrics, including reductions in pain scores and reductions in the incidence of nipple injury. The superiority of these interventions highlights the need to move beyond non-specific interventions to evidence-based approaches in supporting breastfeeding mothers. Current research on breastfeeding-related nipple pain and injury interventions is diverse, reflecting the complexity of the issue and the multitude of factors involved. Among the most commonly studied interventions are topical preparations with anti-inflammatory, antioxidant, moisturizing, bactericidal, and antimicrobial properties, which offer direct skin protection and promote tissue repair (39, 40); nipple shells, designed to reduce friction and provide physical support; laser therapy, leveraging photobiomodulation to enhance cellular regeneration (41); optimized breastfeeding facilitate proper fixation and positioning, which are major causes of pain and injury (42); and educational consultations, which empower mothers with knowledge and practical skills for effective self-management, and education on effective breastfeeding techniques delivered by professional health personnel are both crucial for reducing nipple pain and enhancing breastfeeding sustainability.

The second significant finding of this study was that preventive strategies significantly reduced the severity of nipple pain. In contrast, interventions implemented after the onset of pain showed suboptimal outcomes. This result was consistent with the conclusions of a meta-analysis on interventions to promote exclusive breastfeeding—interventions initiated antenatally and continued postnatally demonstrated significantly greater efficacy than those conducted solely during the postnatal periods (43). Therefore, interventions for nipple pain and injury should prioritize prevention and advance the timing of intervention. Specifically, preventive measures have significantly alleviated the symptoms of pain. These measures include the early application of topical preparations and continuous health education, which enable mothers to acquire the knowledge and skills to manage breastfeeding independently. In contrast, interventions implemented after the occurrence of nipple pain, such as laser therapy, have not significantly improved the pain condition.

However, due to the high within-group heterogeneity in the preventive intervention subgroup, the interpretation of the above conclusions should be more cautious. To explore the sources of heterogeneity, we further conducted a subgroup analysis on intervention duration and found that intervention durations exceeding 7 days might be significantly associated with heterogeneity. When the intervention period is long, multiple factors may contribute to increased heterogeneity: on the one hand, during interventions lasting more than 7 days, there may be differences in specific implementation details across studies; on the other hand, with the extension of intervention time, factors such as changes in participants' compliance and fluctuations in physical conditions may vary across studies, thus affecting the research results.

Among the 18 experiments incorporated into this study, only three reported side effects. One reported the possibility of stinging with the use of laser (31%) (35). One study using breast shells reported areola edema (6.8%), discomfort and unsightly side effects (13.8%) (34). Another study reported feedback from mothers regarding discomfort with use of topical lanolin (6.7%) and breast milk (4.8%) (25). In current research, the reporting of adverse effects across various interventions is widely lacking in systematicity and standardization. Fewer than 20% of studies provide explicit documentation of adverse events, and most of these records remain at the level of symptom descriptions, neither adopting internationally recognized severity grading systems nor providing critical details including duration, and management protocols. This state of reporting has created significant “blind spots in safety signaling”. Despite the meta-analysis revealing no statistically significant difference in side effects between the experimental and control groups, the risk ratio (RR) suggested a discernible trend of elevated side effect risks within the experimental group. This discrepancy highlights the need for further investigation into potential adverse impacts, underscoring the importance of monitoring and reporting side effects in future research to comprehensively evaluate the safety profiles of intervention measures.

## 5 Limitations

This scoping review and Meta-analysis has several limitations that should be taken into account. We focused on reviewing studies in English and Chinese, ignoring those in other languages or in the grey literature.

In addition, in 18 studies, the control groups had various non-specific Interventions, including application of breast milk, use of placebos, and no intervention at all. We designated breastmilk application as the control group rather than a specialized topical preparation for the following rationale: In a cited study, for instance, applying breast milk to the nipples was used as the control group, while olive oil application served as the experimental intervention. The olive oil group had specified dosage requirements, whereas the breastmilk group did not (23). Thus, post-breastfeeding breastmilk use merely capitalized on the natural residual coverage of nipples, rather than constituting a deliberate external intervention. Variations in the non-specific interventions received by control groups across studies may introduce bias. This underscores the importance of reporting control group details explicitly in future research to minimize confounding and enhance the validity of meta-analytic syntheses.

The potential risk of publication bias may be associated with the small number of included studies. Nevertheless, the results of sensitivity analyses still support the robustness of the existing conclusions. Future studies should further include relevant high-quality literatures to reduce the risk of bias.

## 6 Conclusion

In summary, our study results indicate that specialized intervention approaches for breastfeeding-related nipple injury and pain (such as applying topical preparations to the nipples, optimizing breastfeeding postures, and providing counseling and education) are significantly superior to non-specific Interventions. The research also shows that implementing preventive measures before the onset of nipple pain is more effective than intervening after pain occurs. However, due to the limitations of the included studies, it is important to interpret these findings with caution. Well-designed clinical trials should be conducted to further clarify strategies for addressing breastfeeding-related nipple pain and injury.

## Data Availability

The original contributions presented in the study are included in the article/Supplementary Material, further inquiries can be directed to the corresponding authors.
